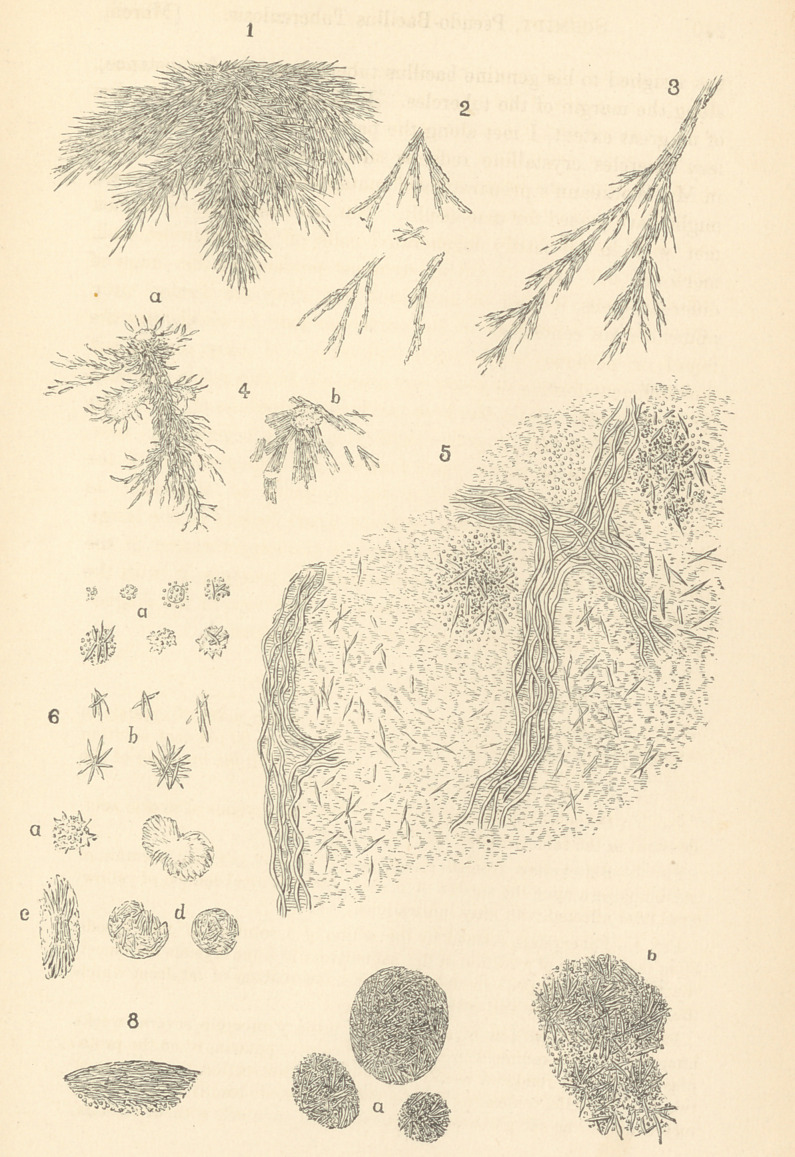# Pseudo-Bacillus Tuberculosis

**Published:** 1883-03

**Authors:** H. D. Schmidt

**Affiliations:** Pathologist to the Charity Hospital of New Orleans


CHICAGO MEDICAL

JOURNAL & EXAMINER.

VOL. XLVI.—MARCH, 1883.—NO. 3.

### Original Communications.

ARTICLE I.

THE PSEUDO-BACILLUS TUBERCULOSIS. By H. D. SCHMIDT, M.D., Pathologist to the Charity Hospital of New Orleans. (Read before the New Orleans Pathological Society.)

In the December number, 1882, of this journal, I published a paper, containing the most prominent points of the results which I had obtained from a series of microscopical investigations into the nature of the so-called bacillus tuberculosis, of Koch. These investigations, as will be remembered, ended in the discovery of minute fat crystals, contained in certain cells of the miliary tubercle, or tuberculous mass, as well as in the expectoration of tuberculous patients.

In this paper, I also mentioned a discovery which I had made, concerning the formation of the same form of crystals in sections of fattily infiltrated or degenerated tissues, when treated with a solution of caustic potassa, or any other alkali; but, for the want of time, I then forebore to enlarge upon this observation. Having, since the above treatise was written, continued my investigations on the crystalline pseudo-bacillus tuberculosis, as well as on the artificial formation of these crystals in fattily infiltrated tissues, I shall now furnish additional details, which may serve to elucidate more fully the facts already published. And, in order to obtain a basis for our discussion upon the formation of fat crystals in animal tissues, we shall, of course, only as far as our subject demands, first refer to some of the facts, observed under the microscope, and relating to the formation of these crystals outside of the tissues, as, for instance, in tallow, or pure human fat.

Before, however, entering upon the discussion of the artificial production of these crystals, a few brief remarks regarding the temperature at which they may be formed, will not be out of place. When, during the month of May or June, of the preceding year, I first discovered that fat crystals would form upon the sections of fattily infiltrated organs, the temperature in this city was quite high, probably ranging from 85° to 90° F., or even more; and, therefore, by treating the sections with a 30 per cent. solution of caustic potassa, I found that the crystals had formed in about twenty-four hours. In a thin film of tallow, spread upon a glass slide and treated with the same solution, I observed a number of single crystals of stearic acid appearing in even as short a time as half an hour, though, for certain reasons, to be mentioned directly, I am now inclined to think that they had previously existed in the tallow, and were simply rendered visible by the potash solution. In sections of a tuberculous lung, treated with this solution, new crystals appeared in the space of two or three days. But, as during the remaining part of the summer, my principal object in view was still to positively determine the crystalline nature of the rod and needle-shaped bodies, formed in the tuberculous cells during life, I neglected to take particular notice of the exact time when the artificially produced crystals would make their appearance in the tissues. Nevertheless, from the numerous sections, which I re-examined from time to time, after being treated with solutions of different alkalies, and of different strengths, I observed that the length of time, required for the artificial formation of these crystals, was proportionate to the strength, not only of the solution, but also of the alkali itself; and, furthermore, that the stronger the alkali, the more perfect and extensive would be the crystallization. In the same manner does the length of time required for the crystallization depend upon the temperature of the surrounding atmosphere, for the lower the temperature, the longer will be the time necessary for the formation of the crystals. With these preliminary remarks, we may now proceed to the consideration of the artificial production of fat crystals, and shall commence with those found in tallow, or human fat, by the action of caustic potassa.

The easiest way to produce these crystals is to spread a thin film of tallow, about one-half an inch in diameter, upon a glass slide, with a clean piece of tallow candle, and, after the application of a few drops of a 30 per cent. solution of caustic potassa, to put on the cover-glass. Then the surplus of the solution is removed by blotting paper, the preparation hermetically closed with Bell's cement, or Canada balsam, and put aside. After one or more days, according to the height of the temperature, minute crystals of stearic acid will successively appear in the film of tallow. Generally, these crystals appear in the form of delicate double-pointed needles, which, by the overlapping of their points, arrange themselves to form filaments, and these again to form branches resembling those of our native pine trees. These branches, finally, represent the components of the most beautiful stellate figures, a portion of one of which is found represented in Fig. 1. But, frequently also, the crystals are met with in the form of minute rods, either single or arranged in small isolated branches, as seen in Fig. 2.

Although I have thus far observed and studied, to a certain extent, the different phases of crystallization through which a patch of fat passes, until it is converted into the above mentioned figures, I have, as yet, not had sufficient time to inquire into the special laws, which determine the particular form of the crystals, or of the different figures formed by them. Besides, the forms which a large patch of fat assumes, while passing through the different phases of crystallization, are quite variable, so that a thorough investigation into the laws governing them could only be made with a considerable sacrifice of time, which I could not make just now. But, as my object of discussing the modes of crystallization of fat, in this place, is to better elucidate the crystalline nature of those minute rods and needles, contained in certain tuberculous cells, I may add that when large neighboring patches of fat pass into the state of crystallization, they generally first approach one another, and unite sufficiently to form a body, the outlines of which resemble those starlike figures ; these, in proportion to the advancing crystallizing process, gradually assume a more definite shape. Upon this body a general mapping out of the branches and needles is then observed, which, as the crystallization advances, becomes more and more distinct, until the individual needles are, finally, completed. Sometimes the body consists of a considerable number of plates, or scales, of various angular forms, arranged in different manners, which are eventually converted into the minute needles or rods. In the case of small patches of fat, or of fat globules, the rods or needles grow directly out of them ; very small fat globules are converted, according to their size, into only one or a few rods. This mode of formation of the rods and needles is, as we shall see hereafter, observed in the sections of tuberculous tissues, some time after having been treated with caustic potassa. Sometimes, the fat crystallizes into a prismatic, or otherwise angular mass, composed of the minute rods.

If a film of tallow upon a glass slide is treated with a solution of sodium hydroxide—of the strength as first prepared by the decomposition of a solution of sodium carbonate with calcium hydroxide—numerous single stearic-acid crystals are produced, which in every detail resemble the pseudo-bacilli tuberculosis; though, here and there, branches, as above described, are also observed to form. The same crystals will be formed, when a film of tallow is treated with *aqua ammoniæ fortior ;* many of them appear here in the form of minute rods, arising from oblong patches of fat, which by mutual communication form a wide-meshed network.

If a film of human fat be spread upon a glass-slide by rubbing the latter with a small piece of adipose tissue, no crystals will form by the action of the potash-solution ; but, if small fragments of the tissue itself are left upon the slide, delicate sword or needle-shaped crystals of margaric acid will appear, forming the same branches and stellate figures as above described in connection with the tallow; among these are also observed plates and prismatic masses.

When the above mentioned crystals, formed in the tallow or human fat, are examined under the microscope with a sufficiently high power,—from 300 to 500 diameters,—it will be observed that they invariably present a very fine, delicate outline on one side, and a very dark and heavy one on the other; these outlines, like those of crystals in general, are very sharply and distinctly drawn. Those stellate figures, as well as single branches, or larger or smaller prismatic masses, formed by these minute fat crystals, present a very brilliant appearance when examined with the polariscope. Upon a dark field they generally appear of a bright yellow, a blue, and a red color. The yellow, however, always predominates; for, though some of the crystalline branches may appear blue, or red, the greater part of the figure still presents the yellow. In changing the field from the dark to the light, by the turning of one of the prisms, these colours, of course, will gradually disappear. Isolated needles, or rods present upon the dark field only the brilliant yellow. Examined with chromatic polarization the crystals exhibit the usual complementary colors. When any group of these crystals is examined with very oblique light, obtained either by means of an Abram's achromatic prism, or by a Power and Lealand's homogeneous achromatic coondenser, they will exhibit the same colors, as upon the dark field of the polariscope, only in a lesser degree.

Having thus briefly discussed the formation of fat crystals, produced by the action of an alkali outside of the tissues, and their behavior when examined with polarized or very oblique light, we may now proceed to consider the formation of these crystals in fattily infiltrated tissues, treated not only with alkalies, but also with certain acids.

Although fat crystals will form in any tissue, affected with fatty infiltration, or degeneration, I have, for the sake of uniformity, made use in these experiments of sections taken from the fatty livers of fatal cases of yellow fever. If, now, a thin section of such a liver is treated with a 30 per cent., or weaker solution of caustic potassa, minute needle or rod-shaped crystals, either single, or forming branches (Fig. 3), or prismatic bodies, will appear in the section in a few days. The individual crystals, as well as the branches they form, closely resemble those formed in the film of tallow; it may therefore be supposed that they represent stearic acid. If such a section be treated with a solution of caustic soda, the same crystals will be observed to appear, only differing slightly in their arrangement in forming branches (Fig. 4). In the same manner are these crystals observed to form, if the section be treated with *aqua ammoniæ fortior.* However, the formation of fat crystals in these sections does not only take place by the action of the above mentioned alkalies, but, moreover, if the sections are treated with formic or glacial acetic acid. When examined upon the dark field of the polariscope they appear in the same brilliant light and colors as the crystals, artificially formed in the tallow by the action of alkalies; and if examined with plain, transmitted light, it will be found that the individual minute crystals resemble in every respect the pseudo-bacilli tuberculosis.

The above described phenomena, relating to the formation of crystals in fat, either pure, such as tallow, or contained in tissues affected with fatty infiltration, or degeneration, through the action of alkalies, or that of formic, or acetic acids, cannot but appear contrary to the known chemical laws, according to which the fat should become saponified by the alkali, without the setting free of its acids. We may, therefore, presume that the liberation of the latter is effected by a combined action of the alkali and the tissues. All alkaline solutions, as we know, affect the protoplasm of most tissues, especially that of the cells; and, if the solution is sufficiently concentrated, it will eventually entirely destroy the protoplasm. The alteration, or final destruction of the tissues, however, can only take place by a chemical combination of the alkali with their albuminous constituents. To explain the phenomenon under consideration, therefore, we may presume that in virtue of this combination, or by a body resulting from it, the glycerine is abstracted from the neighboring fat, and the acids set free to crystalize. The formic and acetic acids affect the protoplasm, especially that of the cells, in a manner similar to the action of the alkalies in a lesser degree; and, very probably, also form a combination with its albumen, leading to the same final results of liberating the acids of the fat in the vicinity. In the case of the tallow, I presume, that though this is generally regarded as pure fat, it still contains some albuminous substances, derived from the tissues from which it was extracted, and with which the alkali would enter into combination; this supposition gains in strength from the fact, above stated, of no crystals being formed in human fat, rubbed upon the slide with a fragment of adipose tissue, and treated with an alkaline solution.

The above explanation regarding the process of the formation of fat crystals in fattily infiltrated tissues by the action of alkaline solutions, of course, rests only upon a mere supposition, to which I was led by observing that these crystals never appeared, before the protoplasm of the tissues was almost entirely destroyed; or, at any rate, before the tissues had completely lost their morphological characters. To render a more detailed and rational explanation of the apparently contradictory phenomena witnessed in this process, must be left to the special chemist.

To the preceding discussion on the artificial production of fat crystals in tallow, or in sections of fattily infiltrated tissues in general, I have now to add some special remarks, concerning the appearance of these crystals in sections of tuberculous tissue, some time after being mounted in a solution of caustic potassa. If such a section be treated with a 30 per cent. solution of this alkali, and examined under the microscope, the first change observed is a general clearing up of the component histological elements, rendering the whole section more transparent. And, as the transparency of the section increases, a number of certain cells, filled with larger or smaller fat globules, associated with, or without black pigment granules, gradually make their appearance. With the increasing action of the alkali on the protoplasm of these cells, they, also, are rendered clearer and clearer, until, finally, their contents are exposed to a full view. It will then be seen that, while all of these cells contain fat globules or pigment, only a number of them may contain the minute fat crystals, or pseudo-bacilli, which I have described and represented in Figs. 1 and 2 of my late paper on this subject. But, as the action of the alkali upon the tissues is still going on, the outlines of the latter are generally lost, until, with the exception of the alveolar elastic tissue framework, they are converted into a shapeless granular substance. While, however, the protoplasm of the tissues, particularly that of the cells, is thus altered, or even destroyed, by the caustic action of the alkali, the fat globules, pigment, and pseudo-bacilli, contained in those peculiar cells, above mentioned, remain unaffected, and are exposed to a better view in the empty fields, mapped out by the elastic-tissue framework. In a section thus prepared, the character of the minute rods and spindles, placed between the fat globules, may be studied to the greatest advantage. The fat globules, left by these cells, whether associated with pseudo-bacilli or not, may always be recognized by their grouping representing the outlines of the cells in which they were formed; very frequently, also, they are associated with pigment. Besides these groups of fat globules, however, a great number of larger or smaller isolated fat globules are now observed over the field of the microscope; these are derived from the fat contained in the remaining more numerous cells of the miliary tubercles, which, also, was set free by the action of the alkali upon the protoplasm of the latter. If, now, the section be examined with the assistance of the polariscope, it will be found that *the pseudo-bacilli, and many of the minute fat globules, between which they lie, appear most brilliantly illuminated, as soon as the prisms of the instrument are crossed, and the field is rendered dark*—while those numerous isolated fat globules remain dark. In fact, the minute rods and spindles polarize the light in such a degree as to remove every doubt that might still exist in regard to their crystalline nature. *True bacilli, such as B. ulna and B. subtilis, as well as all other bacteria, as I know from experience, do not possess double refraction, but remain dark upan the dark field of the polariscope.* If the minute rods, spindles and needles, contained in the cells are very numerous, such as represented in Figs. 7 and 8 of this paper, they will, exactly like the fatty acid crystals found in tallow, exhibit the complementary colors, namely, bright yellow, blue and red. But, it is not alone these minute crystalline rods and spindles that appear brightly illuminated upon the dark field of the polariscope, for, those groups of minute fat globules not associated with pseudo-bacilli, also show the beginning of crystallization by emitting a feeble light which distinctly marks their outlines; very frequently, minute luminous points are observed upon the feebly illuminated area of the group, indicating an advanced phase of crystallization in some of the minute fat globules. The crystallization of the fat into those minute rod, spindle and needle-shaped bodies (pseudo-bacilli), contained in the fattily degenerated cells under discussion, of course, took place during the life of the patient, while the potash solution applied to the section, thus far only served to render them visible by destroying the protoplasm of the cells. But, if now the section be left exposed to the action of the alkali, the fat, set free by the latter in the form of isolated globules, and seen distributed throughout the section, will undergo an artificial crystallization, such as we witnessed in the sections of fattily infiltrated yellow-fever liver, above described. Thus, in the course of several days, if the temperature of the surrounding atmosphere be sufficiently high, a number of minute needle-shaped crystals, arising from the smaller fat globules, will appear distributed over the section, as well as over the rest of the field. These crystals closely resemble, in all their characters, the pseudo-bacilli. Fig. 5 represents a small portion of such a section, several weeks after it had been treated with a potash solution, and when the protoplasm of the cells was destroyed by the action of the alkali. The remains of two of these cells containing the pseudo-bacilli, and of another containing none of them, are seen in the upper part of the drawing; while, scattered over the rest of the section, a number of single minute crystals, artificially formed after the mounting of the section, may be observed.

Very frequently, however, smaller or larger groups of these minute crystals will be observed to arise from the larger fat globules in such a section (Fig. 6 *a* and *b*); in these cases, the process of crystallization generally advances until all the fat of the globule has been converted into the minute crystals. The size of the latter appears to depend, to a certain extent, upon the size of the fat globule; for, if the latter be very small, the crystal will be below the average size. In this manner, those very minute rod-shaped pseudo-bacilli are derived from the minute fat globules associated with them in the respective fattily degenerated cells. Sometimes, a group of crystals arises in mass from a patch of fat (Fig. 6, *c*); or a large fat globule is converted into a cluster of minute, closely packed, rod-shaped crystals, such as seen at *d* of the same figure. If the quantity of fat in such a section be very abundant, large patches of fat, formed by the fusion of a number of large fat globules, will frequently be met with, from which large figures, composed of scales or needles, may be seen to arise. Examined upon the dark field of the polariscope, these crystals exhibit the same complementary colors, as those described before.

Although I have already described, to a certain extent, in my previous paper on this subject, those fattily degenerated cells containing the minute fat crystals, a few additional remarks on their nature and probable origin would not be improper. When a section of a miliary tubercle is examined, while clearing up by the action of the alkaline solution, or of acetic acid, it will be noticed that not all the cells of the tubercle are undergoing fatty degeneration. On the contrary, especially if the tubercle be small and still isolated, only a few cells, filled with fat globules, may be met with, enclosed in the general mass of tubercular cells of one or the other alveolus. While, however, the great majority of tubercular cells present a number of irregular forms, produced by mutual pressure, these fattily degenerated cells may be easily distinguished, not only by their round, or oval form, but moreover, by their opaque, brownish or blackish appearance, caused by the refractive property of the fat globules within; or, also, by the pigment, or the fat crystals which many of them contain. While, however, cells filled with fat globules are met with in tubercles of almost every case of tuberculosis, they do not always contain pigment, or fat-crystals. In fact, as I have before stated, I have met with some cases of tuberculosis, in which I failed to detect any fat crystals in the tuberculous portions of the lungs; while in other cases, great numbers of these fattily degenerated cells were met with, almost completely filled with pseudo-bacilli, minute fat globules and pigment. Some of these cells were very large, while others were comparatively small (Fig. 7). Among these were also observed a number of cells, almost entirely black from the quantity of pigment and minute fat globules which they contained. They were generally found in those tuberculous alveoli bordering on the highly congested, non-tuberculous portions of the lung. Very often, cells, filled with pseudo-bacilli, are observed to adhere to the walls of alveoli, which as yet are not entirely filled up by the proliferating tubercle cells. I am, therefore, inclined to regard them as epithelial cells of the alveoli, which, undergoing fatty degeneration, are incapable of multiplying, and thus become surrounded by the proliferating neoplastic cells during the development of the tubercle. This view is strengthened by the fact that, in many instances, they appear in small groups, lying loose, or simply buried in the tubercular mass; though this view needs to be confirmed by further and more positive observations. In the expectoration of phthisical patients, cells, in every way identical with those above described, are very often met with; they are generally filled with fat globules, though, not unfrequently, they also contain pigment and pseudo-bacilli. In these cases, the expectoration also contains a number of free rod-shaped crystals.

In studying the pseudo-bacilli in sections of tuberculous lung, it is, of course, desirable to have them permanently mounted, both for future study and reference. This, however, is impossible as long as the sections are saturated with the alkaline solution, which not only destroys almost the whole tissue of the section, but, moreover, eventually gives rise to the artificial formation of additional fat-crystals. To mount the sections permanently, therefore, they must be free from every trace of the alkali which they contain. The best method of accomplishing this purpose I have found to be as follows: In order to completely remove the alcohol contained in the sections from the mixture of alcohol and water in which they were kept, they are first put into filtered water, from which they are transferred to a 30 per cent. solution of caustic potassa. In this they are kept for about twenty minuets, when they are again transferred to pure water, in order to get rid of the alkali. From the water they are, by making them float upon a spatula, transferred to the slides, from which the surplus is allowed to run off, while the rest of this fluid is carefully removed up to the margins of the sections, with a camel's-hair brush. Then, two or three drops of the 30 per cent. solution of potassa are put upon each section, and left for about ten or fifteen minutes, at the end of which time the action of the alkali will have rendered the sections sufficiently clear to allow the study of the pseudo-bacilli. Now, the alkali must be removed by repeatedly washing the sections upon their slides with fresh water, until the test-paper shows no alkaline reaction. Then, the water is again entirely removed from the slides up to the margins of the sections, about three drops of a mixture of one part of filtered glycerine and two parts of water put upon each of the latter, and the cover-glasses applied. The preparations may be hermetically sealed with Bell's cement or Canada balsam.

For certain information, relating to the pseudo-bacillus tuberculosis, which I received, after the preceeding pages of this paper were written, I must now slightly deviate from the regular course of our discussion, in order to consider another part of the subject. A few weeks ago, in order to show the correctness of my statements regarding the crystalline nature of the rod-like bodies which I had met with in certain portions of tuberculous lungs, I sent to several medical friends of Chicago and Philadelphia, a number of ready prepared and mounted, as well as unmounted, sections of tuberculous lung tissue, with the special direction of examining them with the aid of the polariscope. Now, the result of these examinations, which I learned several days ago, fully confirms the existence of the pseudo-bacillus tuberculosis which I discovered, but excludes its identity with the bacillus tuberculosis of Koch, which I, accordingly, failed to make visible in the sections of the tuberculous lung. My next object in view, therefore, must be to ascertain the particular cause of my failure, which, as I had very closely followed in the staining of my sections the directions given for the methods of Koch, Ehrlich, and Gibbes, could only be found in the quality of the material which I had used. That this may have been the real cause, I learned, a few days ago, by the meeting of an expert in the staining of tuberculous sputa. This gentleman, Mr. H. Woltmann, of Chicago, had been directed by Dr. Gradle, of the same city, to pay me a visit, on his way to Texas, for the purpose of showing me the veritable bacillus tuberculosis of Koch. Although the time, which this gentleman had to spare was rather short for a thorough examination of the subject, I became, nevertheless, convinced of his familiarity with the staining of sputa by the beautiful preparations he showed me, besides having the pleasure of spending some hours of the afternoon and evening with him in the staining of some of my sections and the examining of the so-called bacillus tuberculosis in his preparations of sputa. Now, as regards the material which I had formerly used in my attempts of staining the parasite, he at once perceived that the aniline oil was impure, and consequently unfit for use; for, while it was of a brown color, it, moreover, showed an acid reaction upon test paper. Fortunately he had with him the aniline oil which he had used for his staining; this was perfectly clear, but, without his previous knowledge, also showed an acid reaction upon the test paper; though it had been alkaline when he first obtained it. Nevertheless, as there was no alkaline aniline oil at hand, he showed me with this his method of staining, which, in substance, is a combination of those of Ehrlich and Gibbes, and which, on the whole, I had closely followed myself. The principal deviation which he makes from these methods, consists in using methyl-blue as the ground color, instead of chrysoidin, for the magenta-stained parasites. For the examination of his preparations of sputa, he had with him one of Zeiss' famous illuminators, which we managed to apply to my Zentmayer's "Grand American" microscopical stand. With this illumination he showed me through my Tolles' $\frac{1}{10}$ dry objective, which possesses a very fine definition, upon the blue ground of the sputa a number of very minute filamentous bodies, appearing thoroughly stained red by the magenta. Though Mr. Woltmann had hitherto looked upon these bodies as representing minute rods, I perceived, at once, the granular appearance of these filaments, which was rendered still more distinct by using a higher eye-piece. Upon my asking for the authority upon which Mr. Woltmann had regarded these filamentous bodies as the veritable bacilli tuberculosis of Koch, he named Dr. Belfield of Chicago, a gentleman who had studied Koch's bacillus in Germany.

Let us now consider a little closer these bodies in Mr. Woltmann's preparation of sputa, which is now by his kindness in my possession. When this gentleman had left, I subjected these filaments to a still closer examination by the use of my Powell and Lealand's homogeneous achromatic condenser, which though not furnishing the intense light obtained by the illuminating apparatus of Zeiss, nevertheless illuminates the object quite sufficiently for an accurate examination. On the contrary, the less intense light, obtained by Powell and Lealand's condenser, is much better suited for the study of the form of these minute bodies than the brilliant illumination obtained by Zeiss' apparatus. This examination still more distinctly showed me that these minute filaments were, in proportion to the difference in the length, composed of from two to six distinct granules; and that, besides, a number of single granules could be seen distributed among them. Though the majority of these filaments were straight, a certain number of them appeared curved. The diameter of the granules composing them I found to be $\frac{1}{1400}$ mill.; while the average length of the filaments is from four to five granules. There remains no doubt that these organisms are identical with those which Gibbes stained and examined in sputa, and which he found to represent rows of spherical bodies (*London Lancet*, August 5, 1882). Like him, I found that they may be well seen with an ordinary objective, such as Bausch and Lomb's, or Zentmayer's $\frac{1}{5}$ student objectives; even the granular character of the filaments may be distinguished by these objectives.

The question may now justly be asked, whether these perfectly stained granular filaments, contained in the sputa, prepared and exhibited to me by Mr. Woltmann, are in reality identical with the bacilli tuberculosis of Koch. Any one familiar with the special character of a bacillus, and particularly with Koch's description of the bacillus tuberculosis, cannot but answer this question in the negative. Bacilli represent minute straight rods with blunt, almost square, extremities; and as such Koch has described the organisms which he discovered in tuberculous lungs. Neither can it be said that the granules, of which the filaments contained in the Mr. Woltmann's preparations of sputa are composed, might represent the spores of the bacillus tuberculosis; for, if these were the spores, we might properly ask, where is the body of the organism? Besides, Koch describes the spores of his bacillus as being oval in form, from two to four in number, and distributed over the whole length of the rod at equal distances. From what I know by my own observations upon the spores of bacillus subtilis, the body of the organism is not annihilated by the setting free of the spores. Therefore, we must conclude that these granular filaments do not represent Koch's bacillus tuberculosis, though they evidently possess the property characteristic of this bacillus, of retaining the particular coloring material which they first absorbed, against another offered to them subsequently. This point, though at present left unexplained, will certainly be cleared up in the end. But the organisms themselves appear to represent sphero-bacteria. I doubt not, but that in many instances these organisms have been stained and passed for Koch's bacilli tuberculosis. They certainly do not represent true bacilli; and I may safely assert that my pseudo-bacilli tuberculosis possess more of the morphological characters of true bacilli than these filaments; particularly, when some of the minute fat-globules are observed to adhere to one or the other crystalline rod which would make the whole resemble a spore-producing bacillus.

From that I have learned from medical journals, hitherto, the examinations made for the detection of the bacillus tuberculosis, were chiefly confined to sputa, for the reason that this matter can be more easily obtained than a tuberculous lung. I, for my part, as I have said before, attach no significance to the presence of micro organisms in the expectoration of phthisical patients, but am the more anxious to behold them in the tissues of the lungs, and in the very place where they are said to give rise to the disease in question. And though I have thus far failed to detect this bacillus in the sections of lung which I stained, I shall certainly take up the subject again as soon as I shall receive the proper material for the staining, and shall not rest until I shall have either satisfied myself as to the existence of this pretended parasite, or, at least have thrown some light upon the different forms under which it appears to different observers.

It is a singular coincidence that my pseudo-bacillus is specially met with in some of the same localities of the lungs which Koch has assigned to his genuine bacillus tuberculosis, as, for instance, along the margin of the tubercles. In a case of miliary tubercles of no great extent, I met along the peripheries of the single miliary tubercles crystalline rods, as small as the stained filaments in Mr. Woltmann's preparation of sputa, and which, at any time, might have passed for true bacilli. But, as these crystals are not met with in all fattily degenerated cells of the tubercles, and, moreover, not even in all tuberculous portions of the lungs of different cases, it may be presumed that they are formed only under certain conditions of the particular part in which they are found, or, perhaps, of the whole individual. I have, as yet, not had sufficient leisure to direct my attention to this point, but shall do so in future. Like the true bacillus tuberculosis of Koch, the pseudo-bacillus, also, together with the fattily degenerated cells, with or without pigment, when found in the expectoration of the patient, may prove valuable in the diagnosis of the case; as their presence must certainly indicate grave lesions in the lungs. The particular quantity of fat, found in the expectoration of the patient, and which may be determined by treating it with the solution of caustic potassa, also, may be of service in the diagnosis, as it will indicate the grade of fatty degeneration.

### EXPLANATION OF ILLUSTRATIONS.

FIG. 1.—Portion of a comparatively large stellate mass of crystals of stearic acid, formed in a film of tallow, treated with a 30 per cent. solution of caustic potassa. The individual crystals, of which the branches of the mass are composed, appear in the form of needles.

FIG. 2.—Branches, formed by minute rod-shaped crystals of stearic acid, met with in the same preparation.

FIG. 3.—Fat-crystals, formed by the action of a 30 per cent. solution of caustic potassa upon the surface of a thin microscopical section of yellow-fever liver affected with fatty infiltrations.

FIG. 4.—Fat-crystals, formed by the action of a solution of caustic soda upon the surface of a section of the same liver; in *a* the crystals are lancet-shaped, in *b* rod-shaped; in these figures a few patches of fat, from which the crystals arise, are still seen.

FIG. 5.—Small portion of a section of miliary tubercle, several weeks after having been mounted in a solution of caustic potassa, when the protoplasm has been rendered nearly invisible. In this section the remains of two of those cells, containing the fat crystals (pseudo-bacilli tuberculosis), together with the fat globules left from another such cell without crystals,

1
2
3
a
4
b
5
a
6
b
a
c
d
8
b
a may be still observed in the upper part of the drawing; while, further below, a number of single needle-shaped crystals are seen scattered throughout the section. The latter were artificially formed, some time after the mounting of the section.

FIG. 6.—Fat-crystals, formed by the action of the potash solution in another section of tuberculous lung, some time after its mounting; *a* represents the crystals while arising from the fat-globules; *b*, different groups of the crystals after the entire consumption of the fat; *c*, the crystals arising in mass from the fat-patches; *d*, large fat globules, entirely converted into minute rod and needle-shaped crystals.

FIG. 7.—Large cells, met with in a section of the lung from a case of pulmonary phthisis (cheesy pneumonia); they are nearly filled with pseudo-bacilli tuberculosis, minute fat-globules and pigment granules; *a*, group of unbroken cells; *b*, a large cell crushed by pressure made upon the cover glass, in order to expose the crystals to full view.

FIG. 8.—One of these cells almost completely filled with the crystals which are arranged parallelly.

## Figures and Tables

**Fig. 1. Fig. 2. Fig. 3. Fig. 4. Fig. 5. Fig. 6. Fig. 7. Fig. 8. f1:**